# Endoscopic management to different isolated maxillary fungal pathologies: case series of a tertiary hospital

**DOI:** 10.1093/jscr/rjaf017

**Published:** 2025-01-23

**Authors:** Maria Alabdulaal, Zahraa Almuhanna, Moath Alfaleh, Ghadeer Bu Saeed, Abdullah Alsaid, Ali Almomen

**Affiliations:** College of Medicine, Arabian Gulf University, Manama 1000, Bahrain; Department of Otolaryngology-Head and Neck Surgery, King Fahd Hospital of the University, Al-Khobar 34445, Saudi Arabia; Department of Otolaryngology-Head and Neck Surgery, King Fahd Specialist Hospital, Dammam 32253, Saudi Arabia; College of Medicine, Imam Abdulrahman Bin Faisal University, Dammam 34212, Saudi Arabia; King Khalid Hospital, Hail 55421, Saudi Arabia; Department of Otolaryngology-Head and Neck Surgery, King Fahd Specialist Hospital, Dammam 32253, Saudi Arabia

**Keywords:** fungal sinusitis, isolated maxillary sinusitis, endoscopic sinus surgery, case series

## Abstract

Isolated maxillary fungal pathologies involve a variety of clinical entities. These include invasive and non-invasive variants, where each has a unique pathogenesis, clinical manifestation, and approach for management. The aim of this case series is to investigate the several ways that fungal infections of the maxillary sinus might present, with the approach to diagnose and manage these conditions. Several discrete maxillary fungal diseases were studied, including fungal ball, acute fulminant invasive fungal sinusitis, allergic fungal sinusitis, and chronic invasive fungal sinusitis at a hospital. For every condition, several treatment options, clinical manifestations, and diagnostic strategies were investigated, which are greatly influenced by the degree of invasiveness as well as the patient’s immunological status. Optimizing patient outcomes, especially in more aggressive types of the disease, requires an early and proper diagnosis. Understanding the various symptoms of these fungal infections is critical for a timely management.

## Introduction

Isolated maxillary fungal pathologies, although uncommon, have increased in the past few years [1]. It refers to a variety of fungal diseases, each having its own pathophysiology, clinical manifestations, and means of treatment. These conditions may manifest in either invasive or noninvasive forms, such as fungal ball (FB), chronic invasive granulomatous and acute fulminant invasive sinusitis [2]. One major risk factor for acquiring such conditions is uncontrolled diabetes [3]. Typical symptoms include nasal discharge, blockage, and facial pain [4]. The diagnosis is based on clinical symptoms, imaging assessments, and fungal cultures. Endoscopic sinus surgery is an effective method of treatment, which often consists of a combination of surgical debridement and antifungal therapy [5].

## Case presentations

### Isolated maxillary fungal ball

**Figure 1:**
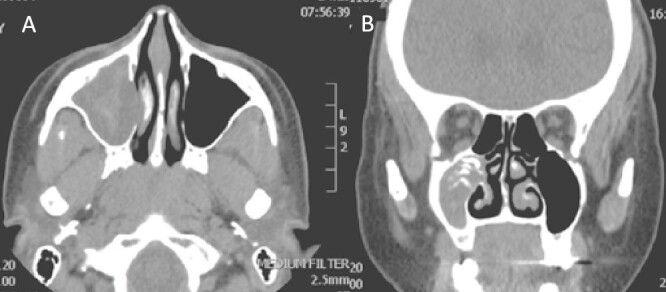
Axial (A) and coronal (B) images of a nonenhanced CT scan of the paranasal sinuses showing right maxillary sinus complete heterogenous opacification.

**Figure 2:**
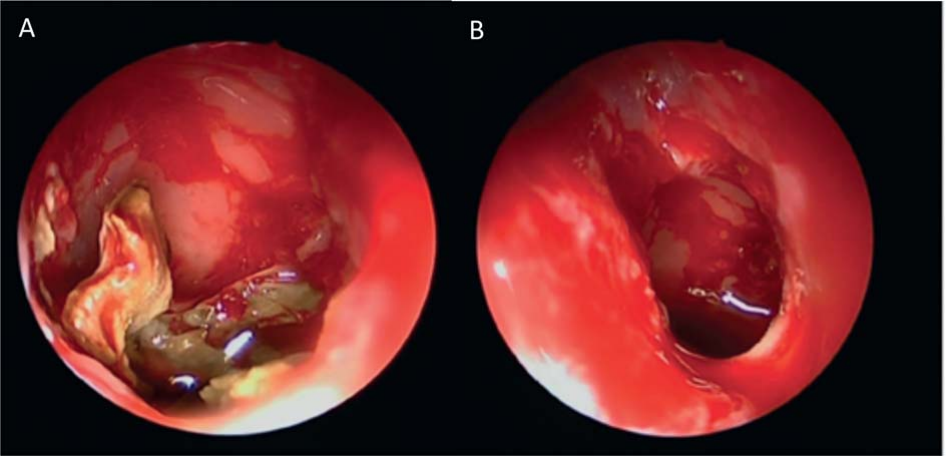
Intra-operative endoscopic view (A) right middle meatal antrostomy showing fungal debris, (B) clean right maxillary sinus after removal of the debris.

A 30-year-old lady presented to the clinic with a complaint of right facial pain, recurrent rhinorrhea with post-nasal discharge, nasal endoscopic examination was unremarkable. A non-contrast computed-tomography scan (NCCT) of the paranasal sinuses (Fig. 1) was suggestive of isolated right fungal maxillary fungal ball (FB). The patient underwent endoscopic sinus surgery (ESS) with (Fig. 2) right middle meatal antrostomy and removal of fungal debris. The patient was followed up regularly and remained symptom free.

### Isolated maxillary acute fulminant invasive fungal sinusitis

**Figure 3:**
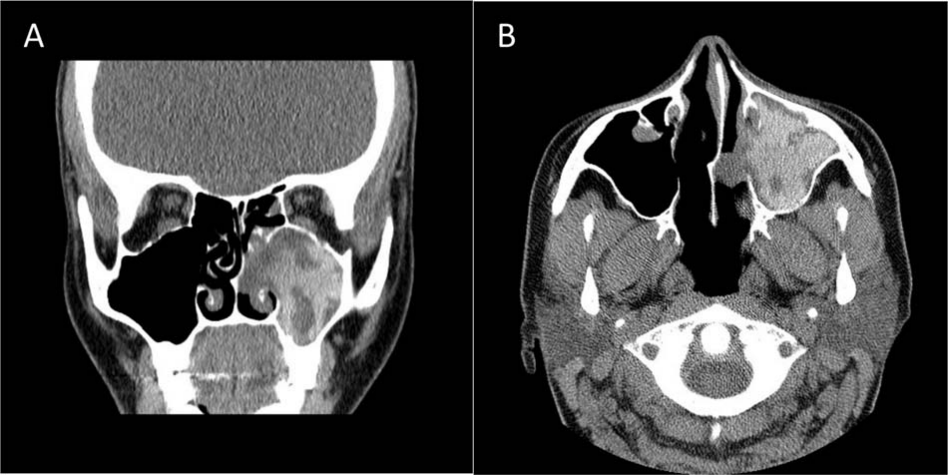
Coronal (A) and axial (B) CECT scan images showing left complete maxillary sinus heterogenous opacification.

**Figure 4:**
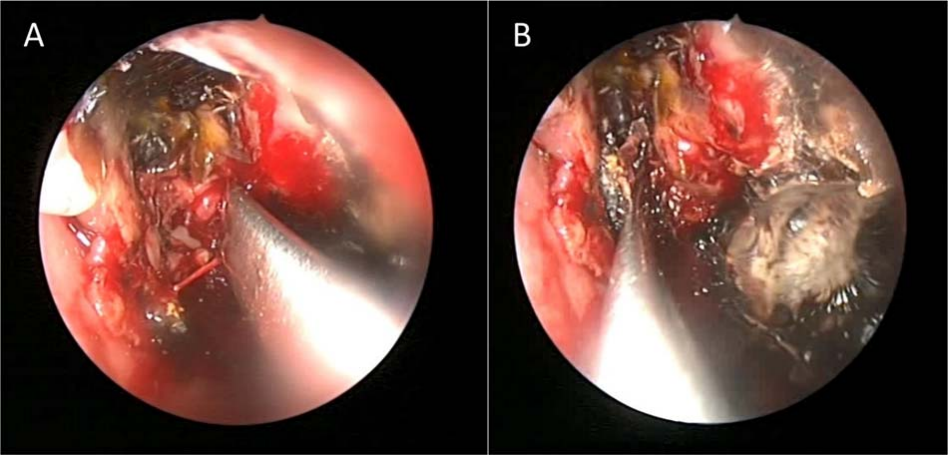
Intra-operative endoscopic maxillary examination (A and B) showing invasive fungal debris.

A 12-year-old young girl with relapsing acute myeloid leukemia presented with fever, headache, and left maxillary facial pain. Her symptoms were associated with blood-tinged nasal discharge. Nasal endoscopic examination showed friable ulcerative mucosa of the left middle turbinate and osteo-meatal complex which raised a clinical suspicion of invasive fungal sinusitis. Thus, a contrast-enhanced CT scan (CECT) (Fig. 3) was necessary which showed enhancing left maxillary sinus opacity suggestive of acute invasive fungal sinusitis. The patient underwent urgent endoscopic medial maxillectomy with removal of invasive fungal debris (Fig. 4) along with debridement of left middle turbinate, medial maxillary wall, and orbital floor. A diagnosis of invasive mucormycosis was confirmed by histopathology. The patient was started on aggressive treatment in the form of intravenous and oral antifungal medications. She was regularly followed up for the next 5 years with no evidence of recurrence.

### Isolated maxillary allergic fungal sinusitis

**Figure 5:**
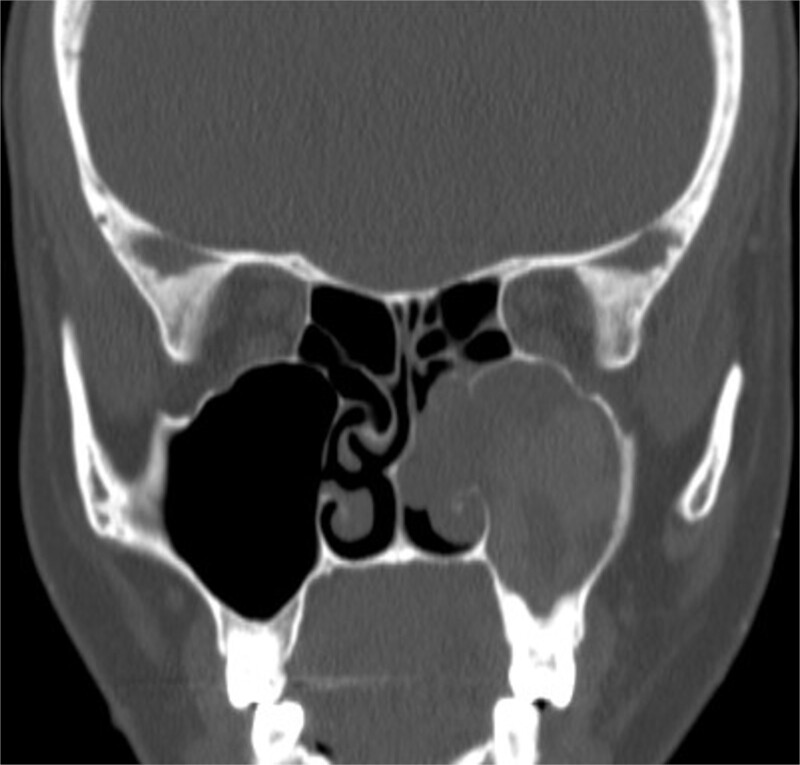
Coronal NCCT image of the paranasal sinuses showing isolated left complete heterogenous maxillary sinus opacification with obstructed left osteomeatal complex.

**Figure 6:**
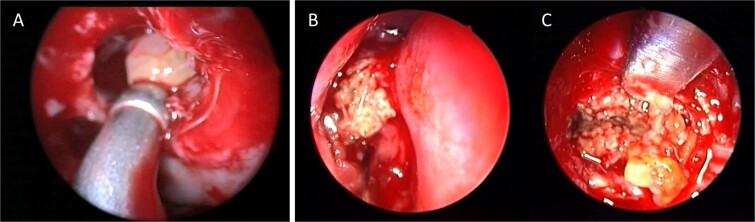
Intra-operative endoscopic view showing (A) polyps in the left osteomeatal complex with (B and C) fungal mud and mucin.

A 37-year-old female with a history of bronchial asthma, complained mainly of chronic headache, left sided facial pain, which was associated with persistent post-nasal drip. Endoscopic examination showed left-sided tenacious discharge filling the middle meatus with grade two polyps. NCCT (Fig. 5) showed left isolated heterogenous maxillary sinus opacity suggestive of allergic fungal sinusitis. She underwent endoscopic sinus surgery (Fig. 6) with removal of fungal mucin and mud, complete left maxillary sinus aeration was ensured. The patient was free of symptoms during her 1 week follow up.

### Isolated maxillary chronic invasive granulomatous fungal sinusitis

**Figure 7:**
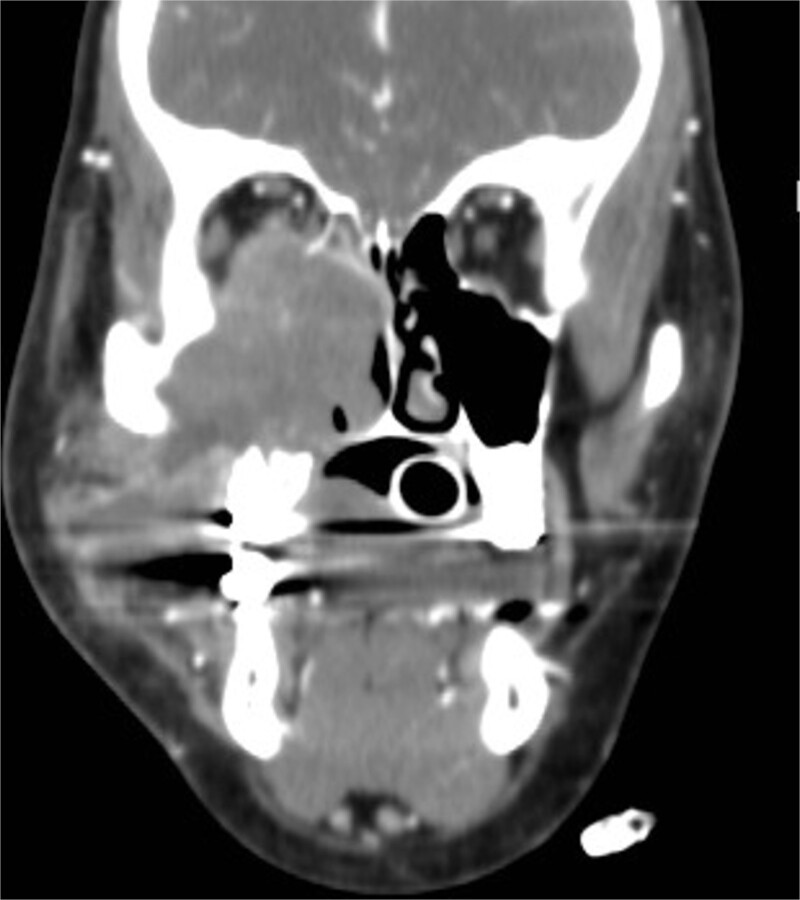
Coronal NCCT image of the paranasal sinuses showing isolated destructive right maxillary sinus lesion violating the right orbit.

**Figure 8:**
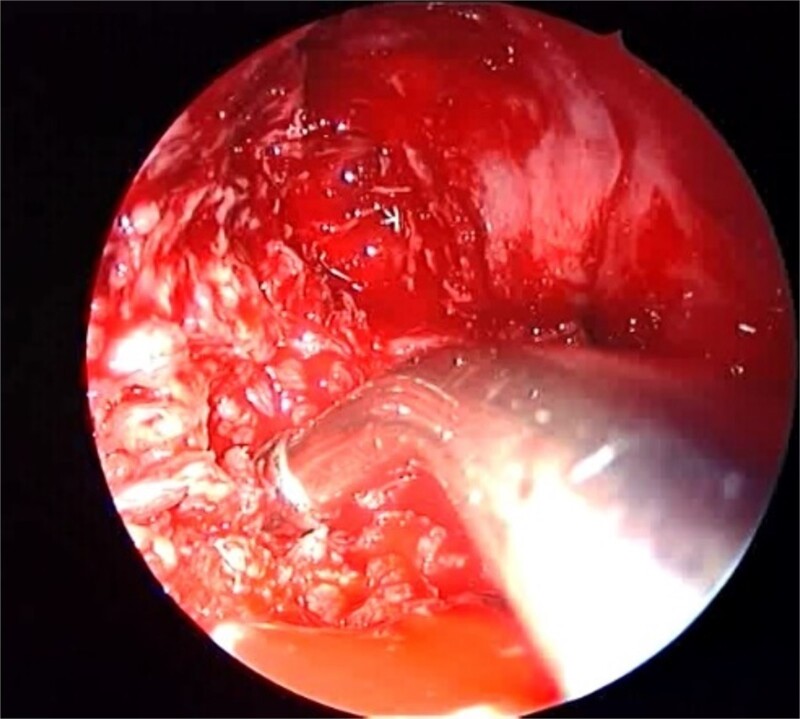
Intra-operative endoscopic view showing invasive fungal granuloma in the right maxillary sinus.

An 18-year-old adolescent mentally challenged male was complaining of chronic right thick purulent nasal discharge, cough, and headache, which did not respond to multiple courses of oral antibiotics. Purulent discharge was seen filling the right nasal cavity and the nasopharynx during endoscopic examination. NCCT images (Fig. 7) revealed a destructive right maxillary sinus lesion. The patient underwent endoscopic sinus surgery, intra-operative frozen section showed granuloma, right medial maxillectomy with removal of the invasive fungal granuloma was performed (Fig. 8), and tissue cultures revealed *Aspergillus flavus*. Infectious disease team was involved, and the patient was started on a 6-months course of oral antifungal agent. The patient remained symptomless throughout his 1 year regular appointments.

## Discussion

Among the paranasal sinuses, the maxillary sinus is affected by a broad range of conditions, each of which brings about particular difficulties for diagnosis and management. Fungal infections of the maxillary sinuses are one of these disorders that have earned considerable levels of interest because of the possibility for significant consequences.

### Fungal ball

The most prevalent non-invasive type that affects the maxillary sinus is FB, sometimes called a mycetoma [6]. This condition usually affects immunocompetent patients and is more common in older adults, with greater incidence in women [7, 8]. In addition, it develops unilaterally without bone invasion as the fungal elements aggregate locally without invading adjacent tissue, usually remains asymptomatic or manifests as chronic nasal blockage [9]. Typically, FB is determined by radiological imaging, which reveals maxillary sinus calcifications. In most of cases, surgical excision using functional endoscopic sinus surgery (FESS) is curative [10].

Given that the pathophysiology of FB in the maxillary sinus is not entirely unknown, endodontic treatment of maxillary teeth has been found to be a significant risk factor in recent studies [7]. The close anatomical closeness of the roots of the maxillary teeth to the sinus floor is probably the cause of this relation, since it might result in the entry of root-filling materials into the sinus during dental treatments [11].

### Acute fulminant invasive fungal sinusitis

Acute fulminant invasive fungal sinusitis (AFIFS), primarily affecting immunocompromised patients, and it is more aggressive fungal infection that can infiltrate the maxillary sinus which typically caused by Mucor species [12, 13]. The pathophysiology of AFIFS determined by fungal spore inhalation, which causes the infection to usually start in the nose and paranasal sinuses [14]. Possible symptoms include fever, rhinorrhea, headache, facial pain, and diplopia. Moreover, mucormycosis can invade orbital and intracranial structures directly or through blood vessels that eventually cause both hard and soft tissues to necrotize [12]. Considering that this fungus invades arteries and might cause thrombosis, it is highly concerning [14]. To prevent the significant morbidity and mortality related to this debilitating condition, early identification, rapid surgical debridement, and systemic antifungal medication, which include intravenous amphotericin B, are essential for ensuring patient survival [13, 15].

### Allergic fungal sinusitis

A prevalent type of fungal rhinosinusitis that primarily affects young individuals and frequently affects the maxillary sinus occurs as allergic fungal sinusitis (AFS) [16]. Nasal polyps and thick, allergic mucus are characteristics of AFS, necessitating their surgical excision as well as corticosteroid therapy [5]. The most common fungus in those scenarios is Aspergillosis [2, 16]. AFS commonly manifests as nasal obstruction, discharge, and face pain; however, localized involvement of particular sinuses, including the maxillary or fronto-ethmoidal regions [2]. Histological investigation, nasal endoscopy, and CT scans are all commonly employed in the diagnosis process [17]. Generally, endoscopic sinus surgery is used for debridement, which is necessary to remove fungal debris from the sinus and allow for the restoration of airflow. Occasionally in combination with antifungal medication [18]. Fungal sinus infections can be treated more effectively if they are identified early and treated promptly. Recurrence rates among AFS patients are relatively high, even with appropriate treatment [5].

### Chronic invasive granulomatous fungal sinusitis

Most cases of chronic invasive granulomatous fungal sinusitis have been documented in Saudi Arabia, Sudan, and India. It is an uncommon disease that usually affects immunocompetent middle-aged people, with *A. flavus* being the most common cause [19]. It usually affects the ethmoid and maxillary sinuses and manifests as a slowly progressing orbital, cheek, or palatal mass with proptosis or sinonasal symptoms. The most common cause is *A. flavus* [19]. Furthermore, the diagnosis is based on histological confirmation of fungal hyphae in tissue and imaging studies [20].

Less invasive surgery is currently the preferred course of treatment, with endoscopic excision followed by azole therapy producing satisfactory outcomes even in more complicated cases [21]. However, the absence of standardized criteria makes diagnosing the condition challenging [20]. The disease may progress readily in immunocompromised people, thus recognizing it early is essential to avoid significant morbidity and mortality [13].

## Conclusion

There are diagnostic and treatment challenges associated with these various isolated maxillary fungal pathologies. The invasiveness and underlying immunological condition of the patient exert a major impact on the treatment and prognosis of isolated maxillary fungal diseases. The unique pathology determines the course of treatment and prognosis for these conditions. FB and AFS are non-invasive conditions that typically respond well to surgical surgery. However, because AFS is recurring, it may need continuous corticosteroid therapy.

On the other hand, more intensive surgical and medicinal treatment is needed for invasive forms, such as acute fulminant and chronic invasive granulomatous fungal sinusitis, for the purpose of assuring long-term recovery as well as avoiding complications. For patient outcomes to be optimized, a diagnosis must be made rapidly and accurately. Therefore, comprehending the diverse manifestations of various isolated maxillary fungal infections is essential for prompt diagnosis and suitable management.
